# A crisis resolution and home treatment team in Norway: a longitudinal survey study Part 2. Provision of professional services

**DOI:** 10.1186/1752-4458-6-14

**Published:** 2012-09-08

**Authors:** Bengt Karlsson, Marit Borg, Stian Biong, Ottar Ness, Hesook Suzie Kim

**Affiliations:** 1 Faculty of Health Sciences, Buskerud University College, Box 7053, Drammen, 3007, Norway

**Keywords:** Crisis resolution, Mental health home treatment, Mental health services, Community mental health

## Abstract

**Background:**

Crisis resolution and home treatment (CRHT) is an emerging mode of delivering acute mental health care in the community. There is a paucity of knowledge regarding the workings of CRHT in the literature. This is the second paper in a series of three from the longitudinal survey of patients of a CRHT team in Norway, which was aimed at describing the characteristics of patients served, professional services provided, and clinical outcomes. This report focuses on the provision of professional services by the team.

**Methods:**

The project was a descriptive, quantitative study based on the patient data from a longitudinal survey of one CRHT team in Norway. The participants of the survey, a total of 363 patients, constituted the complete registration of patients of this team in the period from February 2008 to July 2009.

**Results:**

The average length of service by the team was about 15 days, and those with depression as the major symptom had the longest mean length of stay on the team. The team was engaged in providing a variety of services including individual treatments involving multiple professionals, group treatment meetings, and coordination activities involving external service sectors. While the type of professionals providing individual treatment was not associated with the severity level of clinical problems, those receiving various group treatment meetings had more serious level of clinical symptoms than those not receiving group treatment meetings. In addition coordination activities involving healthcare professionals and social services in the community were in line with the patients' clinical and social needs. The results of the study show that the team functioned effectively in addressing the general guidelines for the functioning of CRHT teams.

## Introduction and background

This paper is the second in a series of three papers presenting findings from a longitudinal survey of a crisis resolution and home treatment (CRHT) team in Norway [1,2]. The survey study is based on the assessment, treatment, and outcome registration data of a total of 363 patients of the team in a period from February 2008 to July 2009. The focus of this paper is on the professional services provided to the patients by the team during this period.

The notion of crisis resolution has been known for the past decades but is only recently introduced as an integral part of community mental health services in Norway. CRHT services are typically associated with the extension of community care to people experiencing mental health crisis, and are also referred to as CRT (crisis resolution team). In line with the national policies and treatment developments in the UK [3-5] the overall objectives of a multidisciplinary crisis resolution and home treatment (CRHT) team are to offer comprehensive treatment and support in people’s home environment and prevent hospital admission. International research and its reviews [2,6-9] reveal variations in the practice of CRHT services, such as team make-up, responsibilities, gate-keeping practices, opening hours, referral procedures, operational practices, and interventions. At the most elaborate level these teams offer full 24-hour services with dedicated psychosocial and medical coverage, and act as gatekeepers to all admissions to in-patient services [7]. CRHT teams function differently from other community mental health teams that have been developed internationally over the last decades. The origins of various community mental health teams are both pragmatic (in the UK) and ideological (in France and Italy), and their objective is to deliver effective multidisciplinary assessment and care to people with mental illness in communities [10]. Community mental health teams refer to various teams with different foci providing a great variety of services and practices. In addition to CRHT teams, community mental health teams include psychosocial rehabilitation teams, early intervention teams (EIT), psychosis teams, case management teams, and assertive community teams (ACT)/assertive outreach teams (AOT), to mention a few [7,10]. The ACT/AOT model was developed specifically to address the needs of service users with severe mental illness or psychosis by providing more intensive and relationally oriented community mental health services by clinical treatment teams that assume 24-hour responsibility [11].

In Norway, a national strategy was formulated to establish a CRHT team at each of the 78 community mental health centers (DPS) [12], and by 2011 there were 59 teams established [13]. A set of guidelines was established based on international experiences with the key service characteristics being defined as (a) provision of assessment and direct care in the context of home and family, (b) working in partnerships with relevant health and social welfare providers, (c) assessment and course of action that may include inpatient treatment, home treatment, crisis resolution by the team, and next-level referrals to health and social services, and (d) brief responding time [12]. Although these criteria describe the way teams are encouraged to work and achieve their targets, the practices seem greatly influenced by local factors, such as allocated resources, interdisciplinary staff make-up, location in terms of urban or rural, and the organizational priorities of the total mental health services in the area [2,9].

As in the UK the CRHT services in Norway also have been developed with a great variation in terms of the modes of operation, staffing, and team make-up [3,12]. It is apparent that the term CRHT connotes different meanings in various contexts. Team profiles and standards of practice vary a great deal, and the term ‘crisis resolution and home treatment team’ conveys a multifaceted and unclear picture [2].

Service characterization and classification of CRHT in the literature offer a heterogeneous picture. However, there are some uniform characteristics such as the objectives of teams typically being associated with preventing hospital admissions, facilitating early discharge, and offering support in peoples’ natural living context during mental health crises. Furthermore teams are characterized by being multidisciplinary (although nurses are the most predominant professionals in many teams), working out of office hours to some extent, and responding rapidly to crisis [3,6,9,14].

In some of the literature value-based as well as acute care competence-based practices are emphasized in describing the core elements of CRHT practices. The practice guidelines for responding to mental health crises published by SAMSHA [15] refer to specific values and principles for an appropriate crisis response. The values highlighted are avoiding harm, intervening in person-centered ways, shared responsibility, addressing trauma, establishing feelings of personal safety, to be based on strengths, seeing the whole person, and emphasizing recovery, resilience, natural support, and prevention. Several principles that can ensure integration of these values in crisis intervention practices are also specified -- such as timely accessing supports and services, providing services in the least restricted manner, available peer support, strength-based plans, and contextual approaches. As revealed in these guidelines, professional practices in acute mental health services are to be person-centered and rights-oriented as well as contextually-oriented both in providing care to individuals and in organizing service provisions. Johnson and colleagues [6] described the current status of consensus of CRHT services for intensive home treatment, drawing on expert sources, in terms of ethical values, theoretical principles, and clinical interventions. Four theoretical principles identified are: (a) hospital admission can be harmful, (b) family and network play critical roles in crisis situations, (c) managing crisis in the community offers skills in coping, and (d) the relationship between a patient and a professional is different in the home from on a ward. Core clinical interventions include the use of strategies for promoting engagement, comprehensive initial assessment, treatment planning, management of risk, symptom management, helping with social and practical problems, starting or adjusting medication, working with families and social networks, and responding to diversity.

In recent research in Norway [2,9,14] it was found that most teams offered services during office hours with some extension to afternoons and weekends, provided family support, met patients without demands for professional referral allowing potential patients to make direct contacts with teams, and provided multidisciplinary services. The literature review on the structure, process and outcome of CRHT services by Sjølie et al. [8] reported that the majority of the published research on CRHT services focuses on structural issues such as cost-effectiveness and admission rates, which have political, economic, and practical implications. However, there is a paucity of research regarding the professional practices of CRHT teams over time, and there is a need to gain insights into the types and characteristics of professional services of CRHT teams.

This paper focuses on two aspects of crisis resolution and home treatment services of a team in Norway: (a) the types of services the team provided to the patients, and (b) relationships between the services provided and clinical assessments made at admission.

## Method

The study applied a descriptive and quantitative design based on client data obtained for a longitudinal study of one CRHT team in Norway. The study was conducted by following a CRHT team that was established in September 2007 for a period of 18 months from February 2008 to July 2009, and the data set included data from all patients admitted to the team during this period.

### Description of the CRHT team and the general protocols for service

CRHT teams in Norway were proposed to increase accessibility to specialized mental health services for patients experiencing acute mental health crisis [9]. The teams were to offer rapid assessment with 24/7 availability, and provide an alternative mode of treatment to hospitalization [9]. The Norwegian mental health system for adults consists of three service levels: (a) at the first level there are primary care physicians and mental health professionals as individual practitioners or teams in primary care settings, (b) at the second level there are community mental health centers of District Psychiatric Service (DPS) for a pre-determined catchment area, which organize service units of inpatient and outpatient services, day-care centres and services, and functional community mental health care teams such as CRHT teams, substance abuse teams, psychosis/rehabilitation/outreach teams, and day/group teams, and (c) at the third level, there are psychiatric hospital wards, including acute wards for in-patient services [9]. People in the community may receive mental health services from private psychiatric mental health professionals in practice in the community, go to outpatient clinics, attend day-care centres, or receive services from various functional teams of the community mental health centers. In each DPS, there are acute hospital beds designated as crisis beds, admission unit beds, open-unit beds, and closed-unit beds. The specific characteristics of CRHT teams are that they are to aim for the resolution of mental health crises in the community, provide services at patients’ homes, respond to patients within a 24 hour period, are organized as multidisciplinary teams, and determine whether or not patients admitted to the team need to be hospitalized. There is no specific guideline regarding the response time to referrers. However, since responses to patients are expected to be carried out within 24 hours, the expectation is that responses to referrers, especially to non-self referrals, to be within a few hours of initial contacts. CRHT teams have been developed to prevent hospitalization of patients who could otherwise be successfully helped in the community by the team. However, CRHT teams do not have the official gate-keeping authority to make hospitalization decisions for all inpatient admissions in communities, only for those who are admitted to the teams.

The CRHT team studied in this research project was established in September 2007 for this district in response to the national mandate for the establishment of a CRHT team in each of the 78 DPS in Norway, and was one of the earliest teams that were established. This CRHT team had a managing director and 12 therapists, including one psychologist, nine nurses and two social workers, who were all prepared to postgraduate level in either psychiatric nursing or mental health work. In addition, one psychiatrist from the DPS worked with the team on a part time basis providing medical services. There was no staff turnover during the study period. The staffing level at the time only permitted the team to operate from 8 am to 10 pm on weekdays and from 8 am to 3:30 pm on weekends. During the opening hours healthcare professionals, patients, family members, and friends were able to make calls directly to the CRHT team for referral. The team was not available 24/7, and did not function formally as the gate keeping unit for psychiatric hospitalizations in the DPS.

The community mental health services of this DPS were organized in the same way as the general configuration for all DPS units in Norway. Neither the data on psychiatric morbidity nor admission diagnoses of psychiatric admissions were available for the DPS; however there were a total of 42 acute psychiatric in-patient beds for the DPS at the hospital, including 1 DPS bed designated as the crisis bed, four acute wards consisting of the admission ward with 6 beds, one open ward with 15 beds, and two closed wards with 10 beds each. Although there were some variations in the ways patients were processed for services by the team, the team followed the general protocol as outlined below:

1. Referral phone call is received from a patient, family member or professional such as primary care physician, private psychiatrist, or nurse.

2. The referral telephone call is screened by the person regarding the appropriateness for admission to the CRHT service, and the screening is discussed and evaluated by the team.

3. As the call is determined to be appropriate for the team’s service, a team member creates a clinical record for this patient to begin the admission process.

4. A meeting is held to assign a team member to this patient.

5. The assigned team member meets with the patient (usually at the patient's residence) in order to assess the crisis situation, fill out the admission registration form that includes an initial assessment, and to decide on intervention plans and further contacts with the patient.

6. The assigned team member continues with the established service plan for the patient.

7. A team meeting is held to decide on a discharge plan.

8. The assigned team member meets with the patient to complete the discharge data form.

9. The team can make decisions regarding hospitalization of patients anytime after their admission to the team. Hospitalization would be one of the discharge destinations for patients.

Therefore, the data for this study were from the patients who were admitted to the CRHT team. A finding from another data set regarding the total number of referral calls received by this team during 18 months from May 2008 to December 2009 was 1,117 of which 418 patients were admitted to the team. We estimate that a similar number of referral calls would have been received by the team during our study period, suggesting that about one third of the referral calls were admitted to the team. There were no data except the basic demographic information on those individuals who were referred but not admitted to the CRHT team. This means that there were no data on the exact nature of communication at the time specifically regarding the reasons for not admitting the patients to the team. However our knowledge of the team suggests that they would have been told to seek other appropriate services in the community such as clinics or day-care centres. Referrals to inpatient psychiatric emergency units would have been done after initial assessments.

### Instruments

A registration form was used to collect the data, and was based on the Multicentre Study on Acute Psychiatry (MAP) [9]. This data form was used to register the CRHT service as a part of a larger study, which included an aggregated data on five CRHT teams in Norway from which a report has been made [2] as well as the patient registration data used in this study. The data set for this study addressed the team’s actual services to the patients, admission assessment, and service duration. The unit of the registration was the patient for our study, with the data obtained at intake and discharge. The data form consisted of eight sections, of which we are reporting on the data from the sections (d), (e), (f), and (h) only in this paper: (a) intake information including referral sources, (b) personal background information, (c) services received prior to the intake, (d) intake assessment, (e) services provided by the team, (f) types of coordination and cooperation contacts made by the team, (g) discharge assessment, and (h) discharge follow-up recommendations. For assessments of patients' mental health status both at intake and discharge, the Health of the Nation Outcome Scale (HoNOS) [16,17] was used. The HoNOS instrument measures severity of mental health problems in the following 12 categories:

1. Overactive, aggressive, disruptive or agitated behavior

2. Non-accidental self-injury

3. Problems with alcohol or substance abuse

4. Cognitive problems

5. Physical illness or disability problems

6. Problems associated with hallucinations and delusions

7. Problems with depressed mood,

8. Other mental and behavioral problems, including ten items (*a = phobia, b = anxiety, c = compulsive behaviors, d= stress/tension, e = dissociative, f = somatoform, g = eating disorder, h= insomnia, i = sexual problem, and j = other problems*)

9. Problems with social relationships

10. Problems with activities of daily living

11. Problems with living condition

12. Problems with occupation and activities.

In this instrument each category is rated in the scale of 0 to 4 with zero for “no problem,” 1 for “minor problem requiring no action,” 2 for “mild problem but definitely present,” 3 for “moderately severe problem,” and 4 for “severe to very severe problem”. For the category #8 that lists 10 items of problems, one major problem is selected for each patient for rating on the same scale of 0 to 4. The scales and subscales of HoNOS [16,17] are HoNOS-Total (HoNOS-T) for summed scores of items #1 to #10, HoNOS-Behavior (HoNOS-B) for summed scores of items #1, #2, & #3, HoNOS-Impairment (HoNOS-I) for summed scores of items #4 and #5, HoNOS-Symptom (HoNOS-S) for summed scores of items #6, #7, & #8, and HoNOS-Social Functioning (HoNOS-SF) for summed scores of items #9 through #12. The HoNOS scale does not measure the level of risk, and neither the information regarding the risk nor the psychiatric diagnoses were available for this study. However, the level of risk can be inferred from the ratings on the categories of *overactive, aggressive behavior* and *non-accidental self injury*.

We constructed a clinical problem grouping from the data, as many patients had more than one problem rated on HoNOS. We categorize the HoNOS scores into two levels: “1” as no clinically significant problem (for the scores of 0 to 2), and “2” as clinically significant problem (for the score 3 or 4) in order to identify co-occurrences of problems. We also grouped the items of “*overactive/*aggressive”, “*problems with alcohol & drug abuse*”, “*cognitive problems*”, “*physical illness or disability problems*”, “*phobia*”, “*compulsive behaviours”,* “*dissociative*”, “*somatoform*”, “*eating disorder*”, and “*other problems*” as a consolidated category as “other problems” for this construction. This was done because there were only few patients on these items with the ratings of 3 or 4, except the item on “*physical illness or disability*” which was viewed to refer to non-mental health problem. The final instrument for the clinical problem type includes seven types labelled as specified in the following:

1. No Problem Type - No clinically significant problem

2. Stress only Type – One problem of stress only (anxiety, stress/tension, or insomnia)

3. Self-harm Type - Self-harm only or with other problems including depression

4. Psychosis Type - Psychotic problems only or with other problems including depression

5. Depression Type - Depression only or with other problems except self-harm and psychotic problems

6. Single Problem Type - One other problem only (Of those categorized as *other problems* in the recode)

7. Miscellaneous Type - Two or more other problems

Because there was no case in which both psychosis and self-harm occurred together, it was possible to anchor psychosis and self-harm as the anchors independent of each other in constructing these types. However, as depression co-occurred with these problems, depression is used as an anchor for combinations other than those with either psychosis or self-harm.

In addition to HoNOS, patients were also rated on the Global Assessment of Functioning scales (GAF) both for symptoms (GAF-S) and functioning (GAF-F) at intake and discharge. GAF is a numeric scale (0 through 100) used by mental health clinicians and physicians to rate subjectively (by raters) the social, occupational, and psychological functioning of adults (e.g., how well or adaptively one is meeting various problems-in-living) [9,18]. Ten ranges of score specify the levels of symptom and functioning ranging from the highest level for no symptoms (GAF-S) and superior functioning in a wide range of activities (GAF-F) to the lowest level for persistent danger of severely hurting self or others (GAF-S) and persistent inability to maintain minimal personal hygiene (GAF-F).

### Data collection procedures

The team members of the CRHT team were trained to use the questionnaire including HoNOS and GAF at the time the team was established. The responsible team member for each patient at admission and discharge filled out the questionnaire. This data collection was done specifically for this research project. The researchers held quarterly meetings with the professional staff of the team in order to re-train their use of the registration form throughout the data collection period. The data were collected on all patients who went through the intake process for the team during the study period.

### Data analysis

The data were analyzed by the statistical software PASW for Windows version 17.0 for SPSS for descriptive statistics. When comparing groups the Student’s *t*-test or F statistics were used for continuous variables, and the Pearson’s chi-square test was used for categorical variables.

### Ethics

The Regional Medical Research Ethics Committee, Health Region II (South) of Norway and the Norwegian Social Science Data Services on behalf of The National Inspectorate approved this study.

## Results

The findings regarding the two research questions addressing the types of services provided and the relationships between admission assessment and services are organized by the general guidelines specified for the functioning of CRHT teams in Norway [8], which include (a) provision of assessment and direct care in the context of home and family, (b) working in partnerships with relevant health and social welfare providers, and (c) assessment and course of action that may include inpatient treatment, home treatment, crisis resolution by the team, and next-level referrals to health and social services, and (d) brief responding time.A total of 363 patients received services from this CRHT team during the 18 months period. This patient group consisted of 65 % females and 35 % males, the majority in the ages of 26 to 64 years (80 %), 43 % married or having a partner while 40 % living alone, and the majority being Norwegian (85 %). About one third of the patients (38 %) referred themselves to the team, while 25 % were referred by primary care physicians. Twenty one percent (21 %) of the group indicated at admission that their current mental health problems were either new or recent onset episodes, while 25 % stated their problems to be recurrences after a period of remission and 54 % indicated that the problems were aggravation of chronic mental health problems. At admission the patients were assessed using the HoNOS, GAF-S, and GAF-F to identify types of clinical and social problems the patients had at the time. From these assessments we found that in general the patients had a moderate level of psychiatric symptoms/problems with depression as the most prevalent problem.

There were five different characterizations of the types of services provided by the team in the data: (a) individual treatment meetings with professional providers of the team, (b) group treatment meetings involving patients/families and/or the members of the team, (c) the type of coordination/cooperation contacts made by the team, (d) psychotropic medication use, and (e) development of care/treatment plans.

### Provision of assessment and direct care in the context of home and family - types of service provision by the team

In response to the patients' needs for mental health care, the CRHT team provided direct care by individual treatment meetings mostly held at patients' homes and group treatment meetings held at the team's office. Individual treatment meetings refer to services provided by the members of the team in addressing patients' crises and problems. As shown in Table 1, the majority of the patients were seen by psychiatric nurses (95 %) and social workers (76 %), while one fourth of the patients met with clinical psychologists and 12 % were seen by psychiatrist. However, the patients often had individual treatment meetings with more than one member of the team. About one fourth of the patients (23 %) were seen only by psychiatric nurses, while nearly one half (46 %) were seen by both a psychiatric nurse and a social worker. On the other hand, about one third of the patients (31 %) were seen by psychiatrist and/or clinical psychologist in addition to nurses and/or social workers. There was no difference in the type of professionals and the combination type of professionals providing individual treatment meetings in terms of the patients' mental health status in the HoNOS categories, the clinical problem types, HoNOS scales, and GAF scales.

**Table 1:** Distribution in the type of professionals providing individual treatment, and the type of group treatment meetings held

	**Service Type**		**N = **	**(%)**
**Professional providers of the team for individual treatment**	Psychiatrist (Missing = 10)	Yes	43	(12.2)
		No	310	(87.8)
		Total	353	(100.0)
	Clinical psychologist (Missing = 9)	Yes	85	(24.0)
		No	269	(76.0)
		Total	354	(100.0)
	Psychiatric nurse (Missing = 2)	Yes	343	(95.0)
		No	18	(5.0)
		Total	361	(100.0)
	Social worker (Missing = 8)	Yes	270	(76.1)
		No	85	(23.9)
		Total	355	(100.0)
**Combination of professional providers for individual treatment**	Psychiatric nurse only		78	(23.0)
	Psychiatric nurse & social worker		155	(45.7)
	Psychiatrist/clinical psychologist & psychiatric nurse/social worker		37	(10.9)
	Psychiatrist, clinical psychologist, psychiatric nurse, and social worker		69	(20.4)
	Total (Missing = 10)		353	(100.0)
**Type of group treatment meetings held**	Group therapy meeting (Missing = 8)	Yes	8	(2.3)
		No	347	(97.7)
		Total	355	(100.0)
	Family/network meeting (Missing = 5)	Yes	133	(37.2)
		No	225	(62.8)
		Total	358	(100.0)
	Team treatment meeting (Missing = 7)	Yes	73	(20.5)
		No	283	(79.5)
		Total	356	(100.0)
**Combination of group treatment meetings held**	No meeting		185	(52.4)
	Family/network meeting only		91	(25.8)
	CRHT team treatment meeting (including group therapy)		40	(11.3)
	Family/network meeting & CRHT team treatment meeting (including group therapy)		37	(10.5)
	Total (Missing = 10)		353	(100.0)

The CRHT team provided various group treatment meetings for the patients such as group therapy meetings involving patients with similar clinical problems with a member of the team (group therapy meeting), family/network meetings involving patients and their family or network members with a team member assigned to specific patients (family/network meeting), and meetings of the team members together with specific patients to address patients' problems as a team (team treatment meeting). As shown in Table 1, about one third of the patients received family/network meetings, and about one fifth of the patients were involved in team treatment meetings. Only a few cases were involved in group therapy meetings. The data also showed that about one half of the patients (52 %) did not receive group treatment meetings, while one fourth (26 %) were in family/network meetings only and about one fifth (11 %) were in team treatment meetings only. About 11 % received both family/network meetings and team treatment meetings.

None of the HoNOS categories by themselves was significantly different according to the type of group treatment meetings held for the patients. However, as shown in Table 2, the means in the HoNOS-Total and HoNOS-Social Functioning according to the type of group treatment meetings held for the patients were significantly different. The group that had the combination of family/network and team treatment meetings had the highest means in these HoNOS scales meaning more severe level of clinical symptoms (HoNOS-T) and more problems in social functioning (HoNOS-SF) in the group. Similarly, the means in GAF-S and GAF-F were significantly different among the types of group treatment meetings held as shown in Table 3. The lowest means in both GAF-S and GAF-F were in the group with the combination of family/network and team treatment meetings, indicating more serious symptoms and lower level of functioning in this group.

**Table 2:** Means, SD, & SE of Mean in HoNOS-Total and HoNOS-social function by the type of treatment meetings provided by the CRHT team

**Type of Treatment meetings provided by the CRHT team**	**HoNOS-Total**				**HoNOS-Social Functioning**			
	**N =**	**Mean**	**SD**	**SE**	**N =**	**Mean**	**SD**	**SE**
**No meeting**	178	9.12	4.55	.341	184	2.20	2.27	.167
**Family/network meeting only**	87	9.82	4.34	.465	90	2.42	2.63	.277
**CRHT team treatment meeting only**	39	10.72	4.74	.759	40	2.83	2.46	.389
**Family/network meeting & CRHT team treatment meeting**	36	12.11	5.43	.906	37	3.65	3.33	.547
**Total**	340	9.80	4.69	.255	351	2.48	2.54	.136
**ANOVA Results**	F = 4.792* (p = .003)				F = 3.72* ( p = .012)			

*Significant p < .05.

**Table 3:** Means, SD, & SE of Mean in GAF-Symptom and GAF-function by the type of treatment meetings provided by the CRHT team

**Type of Treatment meetings provided by the CRHT team**	**GAF-Symptom**				**GAF-Function**			
	**N =**	**Mean**	**SD**	**SE**	**N =**	**Mean**	**SD**	**SE**
**No meeting**	185	49.78	10.79	.792	185	50.03	13.48	.991
**Family/network meeting only**	91	47.78	9.26	.971	91	47.19	12.85	1.347
**CRHT team treatment meeting only**	40	45.88	9.56	1.511	40	44.60	9.59	1.516
**Combination of Family/network meeting & CRHT team treatment meeting**	36	44.72	9.56	1.593	36	43.39	12.57	2.094
**Total** (Missing = 23)	352	48.30	10.26	.547	352	48.00	13.02	.694
**ANOVA Results**	F = 3.650* ( p = .013)				F = 4.139* (p = .007)			

*Significant p < .05.

Bonferroni post hoc tests, given the non-significant Levene statistics for the test of homogeneity of variance for HoNOS-T (p = .479), GAF-S (p = .859), and GAF-F (p = .121), were carried out to examine the group differences in the combination types in treatment meetings by these variables. The ‘no’ meeting group and the combination of family/network and team treatment meetings were significantly different in the means in HoNOS-T (*Mean Diff = −*2.987*, se =* .844, *p =* .003), in GAF-S (*Mean Diff* = 5.062*, se* = 1.849, *p* = .039), and in GAF-F (*Mean Diff* = 6.638*, se* = 2.340, *p* = .029).

### Working in partnership with relevant health and social welfare providers

The team members made coordination/cooperation contacts either by telephone or face-to-face meetings with various healthcare and social service sectors in the community. As shown in Table 4, in about one half of the patients, there were contacts made with family and network members, with primary care physicians in the community, and with psychiatrists or psychiatric services in the community. The family/network coordination contacts were different from the family/network treatment meetings, as the purpose of coordination contacts was principally to gain information about the patients' social situation in order to mobilize resources within the family/network especially in relation to discharge planning while the purpose of the family/network treatment meetings was to help in resolving mental health crises through therapeutic processes involving family/network members. There was a small fraction of the patients (10 %) who did not receive any coordination/cooperation contact service by the team. About 30 % of the patients received coordination/cooperation contact services from only one sector, while the rest of the patients received a various combination of coordination/cooperation contact services.

**Table 4:** Distribution in the type of coordination/cooperation contacts made by the CRHT team

	**Coordination/Cooperation contacts**		**N = **	**(%)**
**Type of Coordination/Cooperation Contact**	Family/Network	Yes	206	(56.9)
		No	156	(43.1)
		Total	363	(100.0)
	GP in the community (Missing = 2)	Yes	192	(53.2)
		No	169	(46.8)
		Total	361	(100.0)
	RN in the community (Missing = 2)	Yes	103	(28.5)
		No	258	(71.5)
		Total	361	(100.0)
	Psychiatrist/psychiatric service in the community (Missing = 3)	Yes	154	(42.8)
		No	206	(57.2)
		Total	360	(100.0)
	Community daycare or hospital (Missing = 3)	Yes	25	(6.9)
		No	335	(93.1)
		Total	360	(100.0)
	Social/Non-medical Services in the community (Missing = 6)	Yes	70	(19.7)
		No	286	(80.3)
		Total	356	(100.0)
**Type of Combined Coordination/Cooperation Contacts**	No Coordination/cooperation contact		35	(9.8)
	Family only		30	(8.4)
	GP/RN only		50	(14.0)
	Psychiatric service only		20	(5.6)
	Social service only		6	(1.7)
	Family & GP/RN		58	(16.3)
	Family & Psychiatric service		22	(6.2)
	Family & Social service		4	(1.1)
	Family, GP/RN, & Psychiatric service		49	(13.8)
	Family, GP/RN, & Social service		15	(4.2)
	Family, Psychiatric service, & Social service		5	(1.4)
	Psychiatric service & GP/RN		22	(6.2)
	Psychiatric service & Social service		2	(0.6)
	GP/RN & Social service		5	(1.4)
	GP/RN, Psychiatric service, & Social service		12	(3.4)
	Family, GP/RN, Psychiatric service, & Social Service		21	(5.9)
	Total (Missing = 7)		356	(100.0)

The type of clinical problems at admission was significantly different according to whether or not the patients received psychiatric coordination/cooperation contacts and primary care physician (GP)/nurse coordination contacts as shown in Table 5. The patients in the 'psychosis' type (68 % of the group) were more likely to receive psychiatric coordination services than other groups, while the patients with no significant clinical problem (32 %) and in the 'miscellaneous' type (32 %) were less likely to receive psychiatric coordination contact service. On the other hand, the groups in the 'single problem' type (55 %) and the ‘depression’ type (51 %) were more likely to receive coordination services with primary care physicians in the community.

**Table 5:** Distribution in the Clinical problem type by Whether or not coordination contacts were made with psychiatric service(s), and with GP and RN in the community

**Clinical Problem Type at Admission†**	**Coordination with community psychiatric service(s)**				
	**Yes**		**No**		**Total**
	**N (%)**		**N (%)**		**N (%)**
**No clinical problem**	35 (32.1)		74 (67.9)		109 (100.0)
**Stress symptom only**	31 (42.5)		42 (57.5)		73 (100.0)
**Self-harm type**	14 (48.3)		15 (51.7)		29 (100.0)
**Psychosis type**	17 (68.0)		8 (32.0)		25 (100.0)
**Depression type**	29 (47.5)		32 (52.5)		61 (100.0)
**Single problem type**	16 (51.6)		15 (48.4)		31 (100.0)
**Miscellaneous type**	7 (31.8)		15 (68.2)		22 (100.0)
**Total** (Missing = 13)	149 (42.6)		201 (57.4)		350 (100.0)
**Statistics**: *χ*^2^ = 14.571* (*df* = 6; p = .024)					
**Clinical Problem Type at admission†**	**Coordination with GP and/or RN in the community**				
	**GP Only**	**RN only**	**GP & RN**	**None**	**Total**
**No clinical problem**	38 (34.9)	9 (8.3)	13 (11.9)	49 (45.0)	109 (100.0)
**Stress symptom only**	25 (34.2)	9 (12.3)	10 (13.7)	29 (39.7)	73 (100.0)
**Self-harm type**	10 (34.5)	4 (13.8)	5 (17.2)	10 (34.5)	29 (100.0)
**Psychosis type**	4 (16.0)	7 (28.0)	7 (28.0)	7 (28.0)	25 (100.0)
**Depression type**	31 (50.8)	6 (9.8)	11 (18.0)	13 (21.3)	61 (100.0)
**Single problem type**	17 (54.8)	5 (16.1)	4 (12.9)	5 (16.1)	31 (100.0)
**Miscellaneous type**	5 (22.7)	3 (13.6)	9 (40.9)	5 (22.7)	22 (100.0)
**Total** (Missing = 13)	130 (37.1)	43 (12.3)	59 (16.9)	118 (33.7)	350 (100.0)
**Statistics**: *χ*^2^ = 40.784* (*df* = 18; p = .002)					

*Significant p < .05.

**†**For this grouping, those with the scores of 3 or 4 in the HoNOS categories were considered to have given symptoms.

Depression as a separate category in HoNOS, when dichotomised into the non-clinically significant depression group (the values of 0 to 2 in the scale) and the clinically significant depression group (the value of 3 or 4), was significantly different by whether or not the patients received family/network coordination (*χ*^2^ = 4.807, *df* = 1, p = .030). Sixty eight percent (54 of the 80 patients) in the clinically significant depression group received family/network coordination contacts as compared to 54 % of those in the non-clinically significant depression group (151 of 281).

### Assessment and course of action including inpatient treatment, home treatment, crisis resolution by the team, and next-level referrals to health and social services

The course of action taken by the team in addressing the patients' mental health crises and problems included the use of psychotropic medication, development of treatment plans, and provision for coordinated care. These were in addition to individual and group treatments, coordination contacts, and discharge referrals to health and social services.

Thirty seven percent of the patients (132 patients) were on psychopharmacological regime with small numbers of patients receiving serum testing for the drugs (4 %) and systematic evaluation for side-effects (9 %). Of those 143 patients who were on psychotropic medication prior to their admission to the team, only 78 patients (55 %) were also on psychotropic medication while on the team. On the other hand, 51 patients (26 %) of 195 patients who were not on psychotropic medication prior to their admission to the team were on psychotropic medication while on the team. The use of psychotropic medication was significantly different according to the clinical problem type at admission as shown in Table 6. Among the seven clinical problem types, those in the ‘depression’ type was the most likely to be on psychotropic medication while those in the ‘single problem’ type and those with no clinical problem were less likely to be on medication.

**Table 6:** Distribution in the use of psychotrophic medication by the Clinical problem type

**Clinical Problem Type at Admission**	**Use of Psychotropic medication**		**Total**
	**Yes**	**No**	**N (%)**
	**N (%)**	**N (%)**	
**No clinical problem**	28 (25.9)	80 (74.1)	108 (100.0)
**Stress symptom only**	32 (44.4)	40 (55.6)	72 (100.0)
**Self-harm type**	10 (35.7)	18 (64.3)	28 (100.0)
**Psychosis type**	11 (42.3)	15 (57.7)	26 (100.0)
**Depression type**	34 (54.8)	28 (45.2)	62 (100.0)
**Single problem type**	6 (19.4)	25 (80.6)	31 (100.0)
**Miscellaneous type**	8 (36.4)	14 (63.6)	22 (100.0)
**Total** (Missing = 14)	129 (37.0)	220 (63.0)	349 (100.0)
Statistics: *χ*^2^ = 20.345* (*df* = 6; p = .002)			

*Significant at p < .05.

**†**For this grouping, those with the scores of 3 or 4 in the HoNOS categories were considered to have given symptoms.

Individual care plans are required by the local authorities in Norway to ensure that patients who receive mental health services in the community are provided with services which meet individuals' needs. In addition, individual care plans are used to tract progress and changes in patients throughout the health care. In our study group, there were 30 patients who had individual care plans at the time of admission established prior to their admission to the team and additional 12 patients had such care plans established by the team. This is a total of 12 % of the patients with individual care plans. However, the CRHT team's assessment of the patients' needs designated 68 patients (19 %) to be in need of individual care plans. Additionally mental health patients who receive care in the community in Norway also may have psychiatric treatment plans and/or crisis care plans established in order to provide the bases upon which mental health care services proceed for patients. Of the 363 patients in the study group, 45 patients (12 %) and 30 patients (8 %) had psychiatric treatment plans and crises care plans respectively prior to their admission, and additional 13 patients (4 %) for psychiatric treatment plans and 12 patients (3 %) for crisis care plans had these plans established while on the CRHT team. There were a total of 79 patients (22 %) who had one or more of these care plans in place. As shown in Table 7, there were significant mean differences in the HoNOS-T, HoNOS-SF, GAF-S, and GAF-F according to whether or not the patients had any care plans, with those having one or more care plans having more severe levels of symptoms and lower functioning.

**Table 7:** Means, SD, & SE of Mean in HoNOS-T, HoNOS-SF, GAF-S and GAF-F by whether or not the patients had care/treatment plan(s) established

**Scales**		**Those with Care/treatment Plan(s)**	**Those without Care/treatment Plan**	***t*-test results**
**HoNOS-T** (Missing = 12)	Mean	9.636 (N=77)	7.828 (N=268)	t = 2.515* (p = .013)
		(N = 77)	(N = 268)	
	SD	5.740	4.878	
	SE of Mean	.654	.298	
**HoNOS-SF** (Missing = 10)	Mean	2.975 (N=79)	2.026 (N=274)	t = 2.892* (p = .005)
		(N = 79)	(N = 274)	
	SD	2.601	2.458	
	SE of Mean	.293	.148	
**GAF-S** (Missing = 11)	Mean	44.96 (N=78)	49.30 (N=274)	t = −3.453* (p = .001)
		(N = 78)	(N = 274)	
	SD	9.592	10.448	
	SE of Mean	1.086	.631	
**GAF-F** (Missing = 11)	Mean	43.54 (N=78)	49.53 (N=274)	t = −4.146* (p < .0013)
		(N = 78)	(N = 274)	
	SD	10.512	13.560	
	SE of Mean	1.190	.819	

*Significant at p < .05.

Through the healthcare reform movement in Norway, there has been an emphasis on the establishment of coordinated practice in which a designation of coordinator for healthcare has been recommended for the continuity of care, and establishment of coordinating groups involving various healthcare providers for patients are encouraged in order to coordinate services provided by various sectors of health and social services. In this study group there were 56 patients who had coordinators in the community healthcare sector prior to their admission to the team and additional 12 patients who had new coordinators established while on the team. Of these 68 patients with coordinators (19 % of the total), there were 31 patients who had coordinating groups in place prior to their admission and 9 additional patients who had coordinating groups established while on the team. About three fourth of the patients did not have coordinators/coordinating groups.

**Figure 1:**
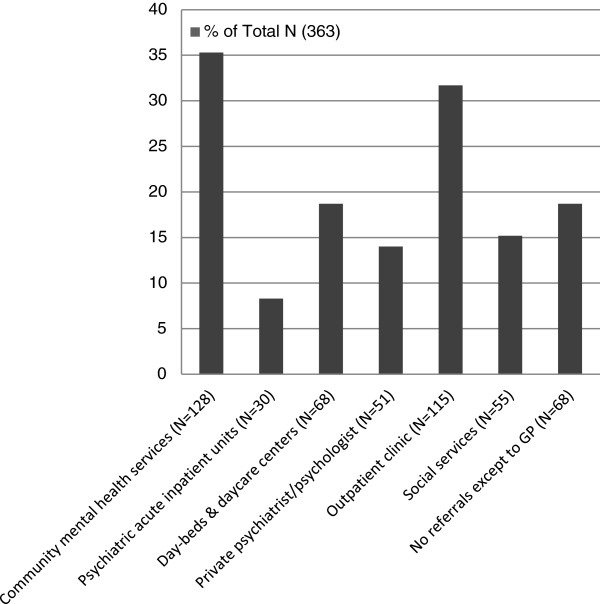
**Number of patients in different follow-up recommendations at discharge (Total N = 363).** Note: Most of the patients had multiple types of follow-up recommendations.

Referrals to health and social services during the course of service are in the form of coordination/cooperation contacts made by the team members. Referrals were also made by the team at the time of discharge. All patients were referred back to their primary care physicians for healthcare. Figure 1 shows the types and numbers of referrals made by the team for the patients as follow-up recommendations upon discharge from the team. Thirty patients (8 %) were recommended for psychiatric inpatient admission, while one third were referred to community mental health services with additional 19 % referred to day-centres or day-beds in the DPS. While 68 patients (19 %) were not recommended for any referral except for primary care physician care, 79 patients (22 %) were recommended for only one referral in addition to the referral to primary care physicians, and 216 patients (59 %) were recommended for more than one referrals.

### Brief responding time

**Figure 2:**
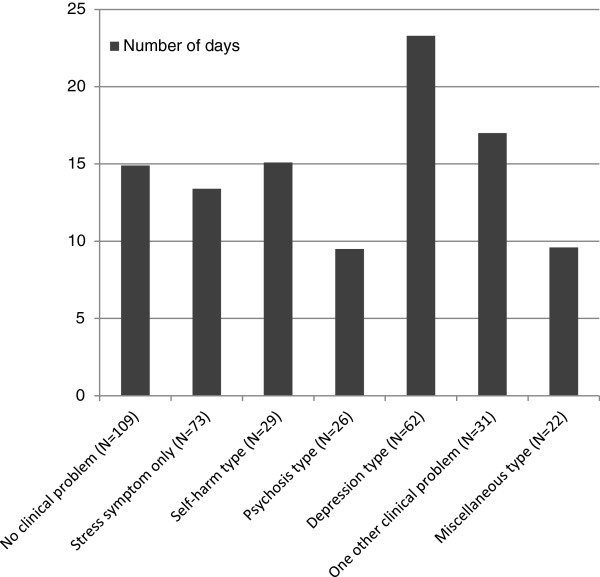
Mean number of days of service on the CRHT team by the clinical problem type at admission.

The average length of stay on the CRHT team service was 15.4 days (SD = 17.227) with a range of zero to 121 days, although 56 % stayed on the service between 1 and 14 days and only 10 % had the length of stay longer than 35 days. As shown in Figure 2, the mean length of stay on the team according to the type of clinical problems at admission was the longest in the 'depression' type (23.3 days) and the shortest in the 'psychosis' type (9.5 days), with the difference among the types statistically significant (F = 3.427, p = .003).

**Figure 3:**
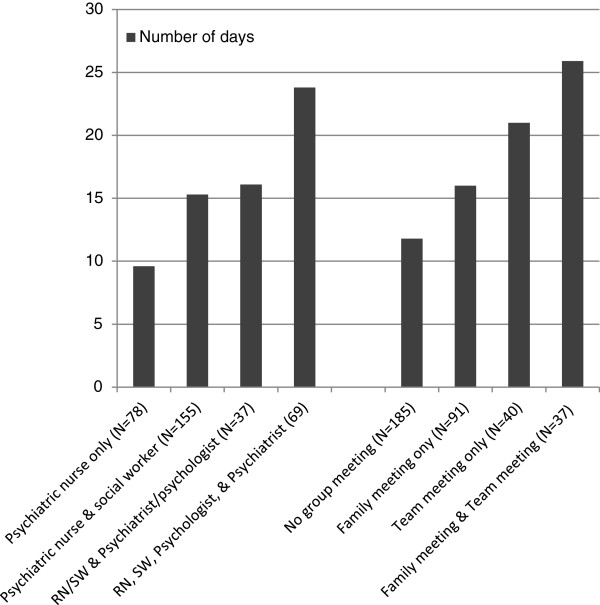
Mean number of days of service on the CRHT team by the type of professionals providing individual treatment and the type of group treatment meetings.

Figure 3 shows the means in the length of stay on the team according to the type of professionals seen for individual treatment and the type of group treatment meetings held. The group that were seen by all four types of professionals had the longest length of stay with 23.8 days, while those seen by psychiatric nurses only had the shortest stay with 9.6 days (F = 8.700, p < .001). The group that had the combination of family/network and team treatment meetings had the longest duration (25.9 days) and the group with no meeting had the shortest duration of 11.8 days (F = 9.185, p < .001).

**Figure 4:**
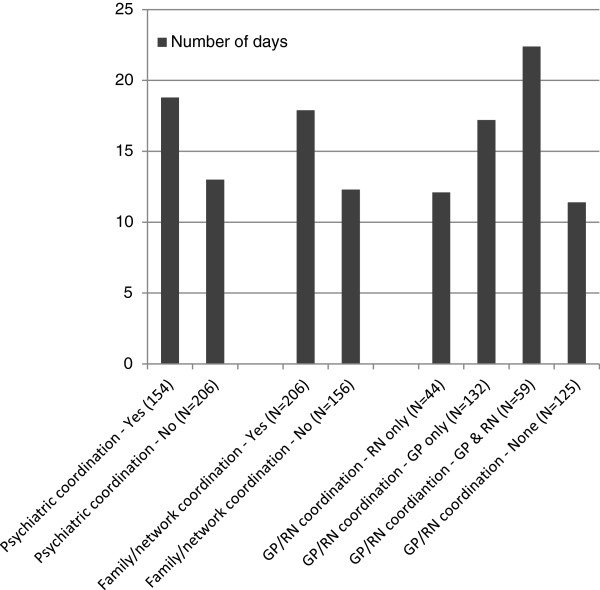
Mean number of days of service on the CRHT team by the type of coordination contacts made by the CRHT team.

The mean lengths of stay on the team according to the type of coordination contacts made by the team are shown in Figure 4. Those with psychiatric, family/network, and GP/nurse coordination contacts had longer stay on the team than those without coordination contacts, and the differences were statistically significant (F = 10.126 with p = .002 for the differences by the psychiatric coordination, F = 9.768 with p = .002 for the differences by the family/network coordination, and F = 6.774 with p < .001 for the differences by the GP/nurse coordination).

## Discussion

The results of our study presented in the preceding section show that there are variations in the services provided by the team and some significant findings in the distributions between the services provided and the problems identified at admission. Since there is no similar report in the literature regarding the comprehensive picture of service provision for mental health care by community-based, crisis resolution service teams, the discussion will follow the general guidelines specified for the functioning of CRHT teams in Norway [8], which include (a) provision of assessment and direct care in the context of home and family, (b) working in partnerships with relevant health and social welfare providers, (c) assessment and course of action that may include inpatient treatment, home treatment, crisis resolution by the team, and next-level referrals to health and social services, and (d) brief responding time.

The first guideline calls for a provision of assessment and direct care in the context of home and family. The results show that the team used various forms of direct care including individual counseling, family/network treatment meetings, and team treatment meetings. The team members were involved in family/network treatment meetings with their patients and team treatment meetings for the patients, suggesting a team-approach. Most of the patients were seen for individual counseling by psychiatric nurses, who made up the majority in the team. About two third of the patients did not meet with a psychiatrist or a psychologist, which was a higher proportion than the findings in a multiple-team study in Norway which was 45 % for the multiple centers [19]. The low level of individual counseling by psychiatrist or clinical psychologist seems to be caused by the team's make-up with one full-time clinical psychologist and one part-time psychiatrist. The effects of this lack of specialized counseling may be significant, although such effects were not analyzed in this study.

As the majority of the patients were seen by more than one professionals for individual counseling, there would have been a great deal of internal consultation among the team members to involve other professionals in individual counseling in response to the case-managers' on-going assessments of their patients. It is apparent that the team used other sources of assessment, such as intake conversations, history, previous experiences, and on-going treatment meetings to guide the ways services were provided to the patients.

One of the key elements for CRHT teams is crises resolution in the context of home and family, based on the idea that home treatment makes it possible to involve family and network in the process of treatment and also to frame plans of treatment in relation to home environment [20,21]. Involvement of family/network members in direct care was evident in this study. More than one third of the patients' family/network members participated in treatment meetings suggesting a high level of involvement of family/network in treatment. However, about one half of the patients received individual counseling treatment only, suggesting either that these patients did not need group treatment meetings or that they did not have family/network members available for meetings. In addition, the team made coordination contacts with more than half of the patients' family/network members, suggesting that input by family/network members were sought to coordinate care.

Since the group that only received individual counseling had better mental health status compared to the group that had the combination of group treatment meetings, it is likely that the patients with more serious mental health problems received various forms of treatment protocols to address their problems. These findings along with the finding that there was no systematic difference in the types of professionals providing individual treatment in terms of HoNOS categories, the clinical problem types, and the HoNOS and GAF scales suggest that clinical assessment at admission may not be as significant in varying the combination of professionals for individual treatment as in applying group treatment protocols. The size of mean differences in the HoNOS scales and GAF scales according to the combinations of group treatment meetings, although statistically significant, may not be clinically significant, suggesting that the level of clinical symptoms and problems of those receiving service from CRHT teams may not vary greatly. Furthermore, the assessment using HoNOS may not be sufficient to direct services in the context of home and family for CRTH teams. A crisis assessment protocol that goes beyond HoNOS is necessary to understand the exact nature of decision making by team members and teams regarding the provision of services.

The second guideline for collaborative practices was upheld in our results. Combinations of multiple professionals providing services within the team and the variety of coordination contacts used by the team in behalf of their patients were evidences assuring that the team has functioned in partnership with health and social welfare providers both within the team and also in the larger service context. The fact that psychiatric nurses were central to the services needs to be emphasized, as it may be conjectured that it was the psychiatric nurses who were responsible for bringing in other professionals of the team into a collaborative service and for making various coordination contacts for patients. Of course the fact that the team was constituted by psychiatric nurses as the majority makes it inevitable for most of the patients to receive services by them. The role for internal coordination played by the nurses in the CRHT team can be viewed in many different ways -- it could be that the role was formally structured within the team, was assumed with an informal understanding, or was a chance occurrence. There is a need for further understanding regarding this role within CRHT teams.

The variety of external healthcare and social service sectors that was used to make coordination/cooperation contacts by the team indicates a commitment to coordinated care and mobilization of resources to meet patients' needs. In addition, the coordination contacts with psychiatrist/psychiatric services and with primary care physicians differently according to the patients' clinical problems indicate that the team's coordination approaches may have been in response to the patients' mental health care needs and problems. However, the small fraction of coordinators and coordinating groups established for the patients suggests the ideal of "coordinated care" within the healthcare system was far from being accomplished.

The findings regarding the third guideline requiring assessment and course of action that may include inpatient treatment, home treatment, crisis resolution by the team, and next-level referrals to health and social services suggest the comprehensive nature of the services by the team. There apparently was a variety of treatments and services provided by the team involving various professional providers. The services provided by the professionals within the team and the extent of coordination contacts made by the team members suggest that the team was able to address this guideline. However, because there is no data regarding the nature of crisis the patients sought services by the CRHT team and regarding the outcomes related to crises, it is not possible to assess how successful the services and processes were in dealing with patients' mental health crises. Since the majority of the patients were discharged from the team's services within 15 days, it is possible to assume that these patients were able to have their mental health crises resolved within this period of time. Assessment measures for mental health crises and outcome measures regarding crises seem to be critical components necessary to gain insights into the effectiveness and efficiency of CRHT teams in this regard. In addition, there was no direct data regarding how and whether a gate-keeping role of the team was implemented, especially in terms of preventing hospitalization. However, since only a very small fraction of the patients were referred to acute psychiatric care in hospitals or in day-care bed services at discharge, it is possible to assume that for most of the patients the mental health crises which were the causes for admission to the team would have been resolved.

The findings regarding the small proportions of the patients with individual care plans and treatment plans are problematic. However, since those with care plans had more serious mental health problems as a group compared to those who did not have them, it can be assumed that efforts were made to establish care/treatment plans for the patients who needed them. As the majority of the patients had long-standing mental health problems, it seems critical for these patients to have individual care plans in place to assure a continuity of care and to assess changes appropriately. It is especially problematic as only one fifth of the patients were considered to need individual care plans by the team. There is a need to study further the processes and protocols involved in developing individual care plans, especially since they are legally required by the local authorities.

With regards to the fourth guideline that CRHT teams are to have brief responding time, our results show that more than half of the patients were discharged within 14 days of admission to the service, with only 10 percent being serviced for longer than 35 days. This is in line with the UK policies [3,5,6], and similar with the findings by Hasselberg et al. [19]. It appears that although the CRHT team functioned with an aim for crisis resolution in a timely manner, there is some evidence that the CRHT team became a more general community mental health team especially for those patients who required multiple services, combined treatment modalities, and multiple sorts of coordination with longer stays on the team. It is possible that mental health crises are not necessarily to be resolved in a short period of time for all patients, but are embedded within patients' on-going mental health problems requiring a long-term surveillance and treatment. This was evident in the findings that most of the patients were referred to community-based mental health care upon discharge from the team. From a policy perspective, and drawing on the data from this team, it may be necessary to consider a next step service module that can be connected to CRHT teams for patients who require continuity and support over time within community specifically in order to prevent recidivism with mental health crises. The findings can be examined further in relation to the core clinical interventions identified by Johnson et al. [6]: (a) use of strategies to promote engagement, (b) comprehensive initial assessment, (c) treatment planning, (d) management of risk, (e) symptom management, (f) helping with social and practical problems, (g) starting or adjusting medication, (h) working with families and networks, and (i) responding to diversity. The results in this study suggest that the team had applied most of these core clinical interventions, although some were only inferable in an indirect manner. The clear evidences of the study were in helping with social and practical problems, starting or adjusting medication, and working with families and networks. The specific behavioral components of team's work that would be associated with the use of strategies to promote engagement, management of risk, and symptom management could not be examined directly in the data that were available. However, it is possible to infer from the findings regarding the involvement of various professionals in individual counseling and the provision of various group treatment meetings that the clinical interventions for the promotion of engagement by the patients and family, management of risk, and symptom management may have been applied. The core interventions regarding comprehensive initial assessment and treatment planning are unknown from the data. There is a need to study further regarding the nature of core clinical interventions and their applications by the team.

Reflecting the results on the official guidelines for the functioning of CRHT teams has provided an insight into the actual workings of the team. However, from the policy perspective and in consideration for establishing standards of practice, there is a need for the guidelines to focus more directly on home treatment as a specific approach in mental health care and on crisis resolution as the major outcome. A requirement for multidisciplinary make-up of CRHT teams also need to be emphasized.

Because there is no comparable data from a longitudinal perspective regarding CRHT teams' services in the literature, it is not possible to assess how this particular team functioned in comparison with other teams. However, it seems the results can be used to formulate initial benchmarks for CRHT services to be applied in assessing other similar teams and services.

This study has several limitations. The lack of generalizability due to its case-study nature is critical. However, the insights the study's results provide can be used to formulate comparative studies and also to rethink the ways standardized data are collected for the workings of CRHT teams. The types of data available and collected for the study have limitations in that the standardized data collection protocol for mental health services in Norway, especially using HoNOS as the basic assessment tool, are limiting for elaboration and for a clear understanding of the processes. There is a need to rethink the standardized protocol for data collection applied to the community mental health services, especially to those with specific mandates such as CRHT teams. Because of the nature of the data used for the study, it was not possible to examine what sorts of mental health crises brought the patients to the team and how the team responded to the crises in a quantitative manner. In terms of data collection procedures used in this study, it is possible that there was some bias in the use of HoNOS as different team members were responsible for making the assessments. Although all team members were trained in the use of HoNOS, a rater-bias would have been possible, and there was no reliability check carried out in the study in this regard. Because of the quantitative nature of the data it was not possible to investigate the characteristics of the processes through which the services were provided to the team's patients. It was impossible especially to gain an insight into how specific values and principles appropriate for crisis response in mental health care have been incorporated into the processes of the team's services.

## Conclusions

The results of this study focusing on the services provided by the CRHT team during a period of 18-months suggest that the team generally met the mandates for the functioning of CRHT teams. The CRHT team used various combinations of services, treatment meetings, and coordination contacts. Although the results are from one specific team, it is possible to use the findings as an initial basis for developing a model of participatory, collaborative practice in CRHT teams.

It appears that psychiatric nurses, as they make up the majority of CRHT teams, may play the key role not only in the provision of crisis-resolution services but also in coordinating services among the team members and with external service sectors. The dynamics in the provision of services by the team may be greatly influenced by the constituting professionals of teams. It is therefore critical to gain further understanding regarding the roles of team members and decision-making protocols in place in the practice of CRHT teams. In future studies, it is necessary to examine the effects of different multidisciplinary make-up of CRHT teams on the type of services provided, application of core clinical interventions, and patient outcomes.

The results that the quantitative assessment measures had a limited role in determining the types of services provided by the team suggest that there may have been other types of assessment information used to make decisions regarding services. There may be data collected by CRHT teams using assessment scales for suicide, safety, etc., and assessment interviews especially in terms of crisis and psychiatric assessment in addition to those used in this study. For further studies, it is critical to gain access to such information in order to gain clearer understanding of the processes used in CRHT teams for the provision of their services. There also is a need to have an extensive qualitative investigation into the workings of CRHT teams and team members’ practice in order to gain insights and knowledge regarding the processes of practice that are applied in crisis resolution in particular as well as mental health care in general especially in relation to the values and principles appropriate for crises care in mental health.

## Competing interests

The authors declare that they have no competing interests.

## Authors' contributions

All authors were actively involved in the research project and contributed to all aspects in the preparation of the manuscript.
